# Incidence, risk factors, and outcomes of ovarian metastasis in early-stage cervical cancer: a population-based analysis of 983 patients

**DOI:** 10.1007/s00404-025-08246-6

**Published:** 2026-01-28

**Authors:** Paolo Gennari, Dennis Luft, József Mészáros, Atanas Ignatov

**Affiliations:** Department of Gynecology and Obstetrics, Otto-Von-Guericke University, Gerhard-Hauptmann-Str. 35, 39108 Magdeburg, Germany

**Keywords:** Cervical cancer, Ovarian metastasis, Adenocarcinoma, Histology, Real-world data, Oophorectomy

## Abstract

**Purpose:**

To assess the incidence, risk factors, and prognostic impact of ovarian metastasis in early-stage cervical cancer using a large population-based registry.

**Methods:**

We retrospectively analyzed 983 patients with cervical cancer classified as pT1a1–pT2b according to the TNM system treated with primary surgery and bilateral oophorectomy. The association between clinicopathological variables and ovarian metastasis was evaluated using Chi-square tests and binary logistic regression. Survival outcomes were assessed with Kaplan–Meier curves and Cox regression.

**Results:**

Ovarian metastases were identified in 0.8% of cases (*n* = 8). Histologic subtype was significantly associated with ovarian metastasis (*p* = 0.010). In multivariate logistic regression, adenocarcinoma histology was an independent predictor of metastasis (OR 9.94, 95% CI 1.99–49.6, *p* = 0.005). Patients with ovarian metastases had significantly worse disease-free and overall survival (*p* < 0.001). Due to the rarity of events, multivariable survival analysis incorporating treatment parameters was limited.

**Conclusion:**

Ovarian metastasis is rare in early-stage cervical cancer but associated with significantly impaired prognosis. Adenocarcinoma histology was independently associated with ovarian metastasis and may be considered when discussing ovarian preservation, although validation in larger cohorts is warranted. These findings support the individualized selection of patients for ovary-sparing surgery.

## What does this study add to the clinical work?

**Table:** 

Ovarian metastasis in early-stage cervical cancer is rare but strongly associated with impaired survival. Adenocarcinoma histology independently increases the risk and should be considered when counseling patients about ovarian preservation.	

## Introduction

Ovarian metastasis from primary cervical cancer is considered an infrequent event, particularly in early-stage disease, yet its occurrence carries critical implications for surgical planning and fertility preservation [1, 2]. While bilateral salpingo-oophorectomy remains a routine component of radical hysterectomy in many cases, the contemporary shift toward individualized, less invasive treatment strategies—especially in premenopausal women—has renewed interest in understanding the precise risk of ovarian involvement across histologic subtypes and stages [3, 4].

Previous studies, including large retrospective cohorts and meta-analyses, have reported overall rates of ovarian metastasis ranging from 1.5 to 3.6% in surgically treated cervical cancers, with adenocarcinoma histology emerging as a consistent risk factor [5–7]. Nevertheless, much of this evidence stems from institutional case series or multicenter datasets with limited generalizability, lacking the population-based perspective necessary for definitive guidance [8].

To inform clinical decision-making in the context of ovarian preservation, particularly in younger women and those with adenocarcinoma histology, robust real-world data are needed [9]. The use of comprehensive tumor registries allows for a broader, more representative analysis of risk factors and survival outcomes across an unselected population [10].

In this population-based study, we aimed to quantify the frequency and prognostic significance of histologically confirmed ovarian metastases in women with stage T1a1 to T2b cervical cancer, as classified by the Tumor–Node–Metastasis (TNM) staging system, who underwent primary surgical treatment including bilateral salpingo-oophorectomy. We further assessed whether adenocarcinoma histology independently predicts ovarian spread and evaluated its impact on overall and disease-free survival.

## Materials and methods

### Study design and patient selection

We conducted a retrospective cohort study using data from the population-based Tumor Registry of Sachsen-Anhalt, Germany. The registry includes comprehensive clinical, pathological, and treatment-related data on all incident cancer cases within the federal state. Women diagnosed with primary cervical cancer between January 1, 2000, and December 31, 2023, were screened (*n* = 7043). The Tumor Registry Sachsen-Anhalt is a state-wide, legally mandated, population-based registry with > 95% case capture and regular external validation, ensuring high data completeness and reliability.

**Fig. 1:**
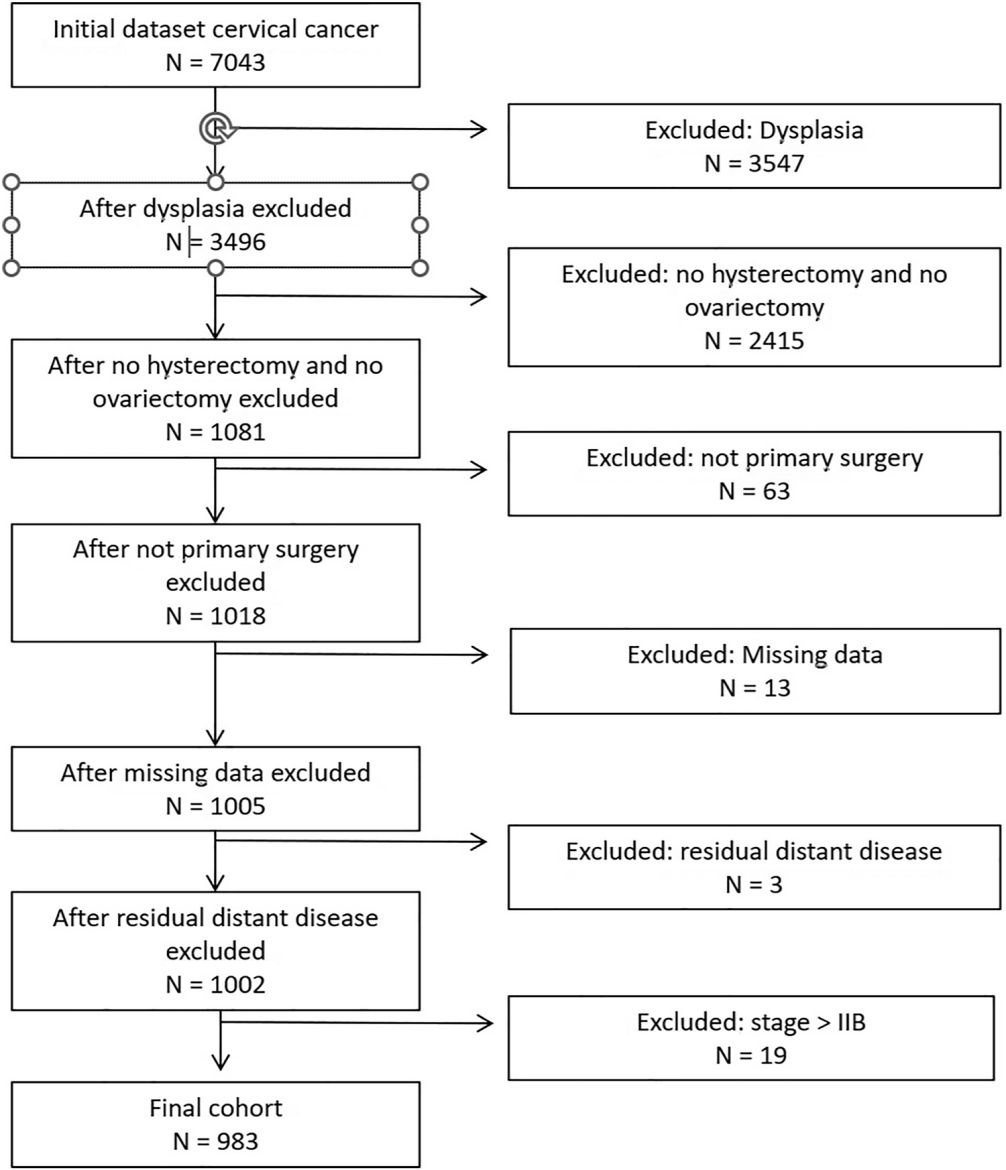
Flowchart of patient inclusion and exclusion from the cervical cancer cohort

Eligible patients fulfilled the following inclusion criteria: histologically confirmed cervical carcinoma with tumor stage pT1a1 to pT2b according to the Tumor–Node–Metastasis (TNM) classification, primary surgical treatment including hysterectomy and bilateral salpingo-oophorectomy, no evidence of synchronous or prior malignancy, and complete clinicopathologic and follow-up data. We used TNM staging rather than the staging system of the International Federation of Gynecology and Obstetrics (FIGO) to avoid automatic upstaging of patients with ovarian or lymph node metastases, which would have excluded them from FIGO stage I–II and biased the analysis. To isolate the prognostic impact of ovarian metastases, we excluded patients with distant metastases to sites other than the ovaries at the time of diagnosis. After applying all inclusion and exclusion criteria, 983 patients were included in the final analysis (Fig. 1). Because only patients undergoing bilateral oophorectomy were included, selection bias cannot be excluded and generalizability to ovarian preservation is limited.

### Data collection

Demographic and clinical variables were extracted, including age at diagnosis, histologic subtype, FIGO stage, TNM stage and presence of lymphovascular space invasion (LVSI). Histologic subtypes were categorized as squamous cell carcinoma, adenocarcinoma, adenosquamous carcinoma, neuroendocrine carcinoma, and other rare variants. Ovarian metastases were identified based on histopathological examination of resected adnexal specimens.

### Endpoints

The primary endpoint was the frequency of histologically confirmed ovarian metastases. Secondary endpoints included overall survival (OS) and disease-free survival (DFS). OS was defined as the time from diagnosis to death from any cause, and DFS as the interval from diagnosis to first documented recurrence. Patients without events were censored at last follow-up.

### Statistical analysis

Categorical variables were compared using the Chi-square test or Fisher’s exact test, as appropriate. A binary logistic regression model was used to assess the association between histologic subtype (categorical variable) and ovarian metastasis, using squamous carcinoma as the reference category. Odds ratios (ORs) with 95% confidence intervals (CIs) were calculated. Due to the low number of events in some subgroups, estimated odds ratios for certain histologic types should be interpreted with caution.

Survival probabilities were estimated using the Kaplan–Meier method and compared using the log-rank test. All statistical analyses were performed using IBM SPSS Statistics for Windows, Version 28 (IBM Corp., Armonk, NY, USA). A two-sided *p* value < 0.05 was considered statistically significant.

## Results

A total of 983 women fulfilled the inclusion criteria and constituted the analytic cohort. The median age at diagnosis was 50 years (range 22–89; mean 51.6 ± 13.2 years). Histologically, 657 patients (66.8%) had squamous cell carcinoma, 232 (23.6%) had adenocarcinoma, 26 (2.6%) had adenosquamous carcinoma, 4 (0.4%) had neuroendocrine carcinoma, and 64 (6.5%) harbored other rare subtypes. Bilateral salpingo-oophorectomy revealed histologically proven ovarian metastasis in eight patients, corresponding to an overall prevalence of 0.8%.

In the univariate analysis, histologic subtype was the only variable that showed a statistically significant association with ovarian spread (*χ*^2^ = 13.282, *p* = 0.010). Metastases were identified in 6 of 232 women with adenocarcinoma (2.6%), in 1 of 657 women with squamous carcinoma (0.15%), and in 1 of 94 women with the remaining histologies (1.1%). No significant relationship was observed for any of the other examined factors. Lymphovascular invasion was present in 6 of 136 metastasis-positive cases (4.4%) versus 2 of 847 metastasis-negative cases (0.24%), but this difference did not reach statistical significance (*p* = 0.718). Likewise, T stage (1a1–2b) was not linked to risk of ovarian metastasis (*χ*^2^ = 4.638, *p* = 0.591); although most metastases occurred in TNM stages pT1b1 and pT2b, the overall distribution across stages did not differ from that of tumor-free patients. Finally, lymph node status (pN) (*p* = 0.996) and age at diagnosis dichotomized at 40 years (*p* = 0.920) showed no measurable impact (Table 1).

**Table 1:** Univariate associations between clinicopathologic variables and ovarian metastasis

Variable	Ovarian metastasis *n* (%)	No ovarian metastasis *n* (%)	*p* value
Age at diagnosis > 40 year	6 (75.0%)	746 (76.5%)	0.920
Histologic subtype			0.010
Squamous carcinoma	1 (12.5%)	656 (67.3%)	
Adenocarcinoma	6 (75.0%)	226 (23.2%)	
Adenosquamous carcinoma	0 (0.0%)	26 (2.7%)	
Neuroendocrine carcinoma	0 (0.0%)	4 (0.4%)	
Other/rare tumors	1 (12.5%)	63 (6.5%)	
TNM stage (pT1a1–pT2b)			0.591
pT1a1–pT1a2	1 (12.5%)	146 (15.0%)	
1b1–2b2	3 (37.5%)	587 (60.2%)	
2a–2b	4 (50.0%)	242 (24.8%)	
Lymph node status (pN)			0.996
Negative	6 (75.0%)	758 (77.8%)	
Positive	2 (25.0%)	175 (17.9%)	
Lymphovascular invasion (L)	2 (25.0%)	352 (36.1%)	0.718

Chi-square or Fisher exact test as appropriate

A binary logistic regression model including histologic subtype as a categorical variable was fitted to assess the association with ovarian metastasis. Histologic subtype was included as a categorical predictor in the model (657 squamous, 232 adenocarcinoma, 26 adenosquamous, 4 neuroendocrine, and 64 rare histologies). Compared to squamous carcinoma, adenocarcinoma was significantly associated with increased odds of ovarian metastasis (OR 17.4, 95% CI 2.1–145.4, *p* = 0.008) (Table 2). No significant association was observed for adenosquamous carcinoma, neuroendocrine carcinoma, or rare histologies, likely due to small subgroup sizes and the overall low number of metastatic events.

**Table 2:** Binary logistic regression analysis of histologic subtype and ovarian metastasis

Histologic subtype	OR (Exp(*B*))	95% CI for OR	*p* value
Squamous carcinoma	Reference	–	–
Adenocarcinoma	17.42	2.09–145.44	0.008
Adenosquamous carcinoma	~ 0.00	–	0.999
Neuroendocrine carcinoma	~ 0.00	–	0.999
Other/rare histologies	10.41	0.64–168.49	0.099

Logistic regression with squamous cell carcinoma as reference. Only adenocarcinoma showed a statistically significant association with ovarian metastasis

### Adjuvant therapy

Among the eight patients with ovarian metastasis, five received combined chemoradiation, one received radiotherapy alone, and two received no adjuvant therapy. In contrast, 975 patients without ovarian metastasis had a more variable treatment distribution: 231 (23.7%) received combined chemoradiation, 150 (15.4%) received radiotherapy alone, 32 (3.3%) received chemotherapy alone, and 562 (57.6%) had no adjuvant treatment (Table 3). Adjuvant therapy was administered with curative intent according to institutional or guideline-based protocols. Only treatments administered within the standard postoperative adjuvant window were included in the analysis. Treatment modalities are summarized in Table 3.

**Table 3:** Distribution of adjuvant therapy by ovarian metastasis status

Ovarian metastasis	No adjuvant therapy	RT only	CTX only	RT + CTX	Total
No	562 (57.6%)	150 (15.4%)	32 (3.3%)	231 (23.7%)	975
yes	2 (25%)	1 (12.5%)	0 (0.0%)	5 (62.5%)	8

RT, radiotherapy; CTX, chemotherapy

### Survival analysis

#### Overall survival (OS)

**Fig. 2:**
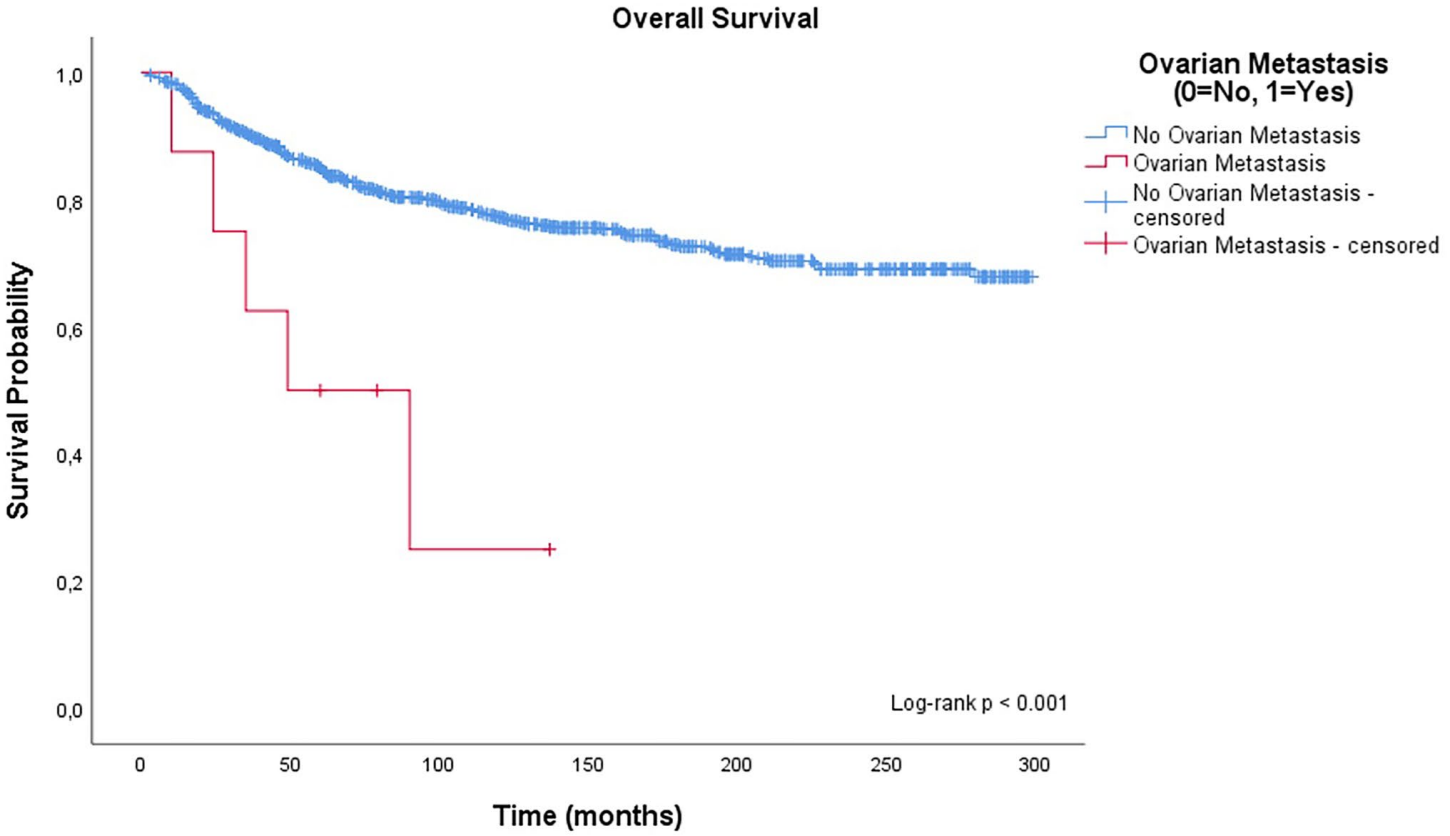
Kaplan–Meier curve for overall survival stratified by ovarian metastasis status. Survival differed significantly between groups (log-rank *p* < 0.001)

Over a median follow-up of 105 months (range 0–299 months), 227 patients died. Patients with ovarian metastases experienced significantly worse overall survival compared to those without metastasis (62.5% vs. 22.8% mortality, respectively). Kaplan–Meier curves demonstrated a pronounced survival disadvantage in the metastasis group (log-rank *p* < 0.001; Fig. 2).

#### Disease-free survival (DFS)

**Fig. 3:**
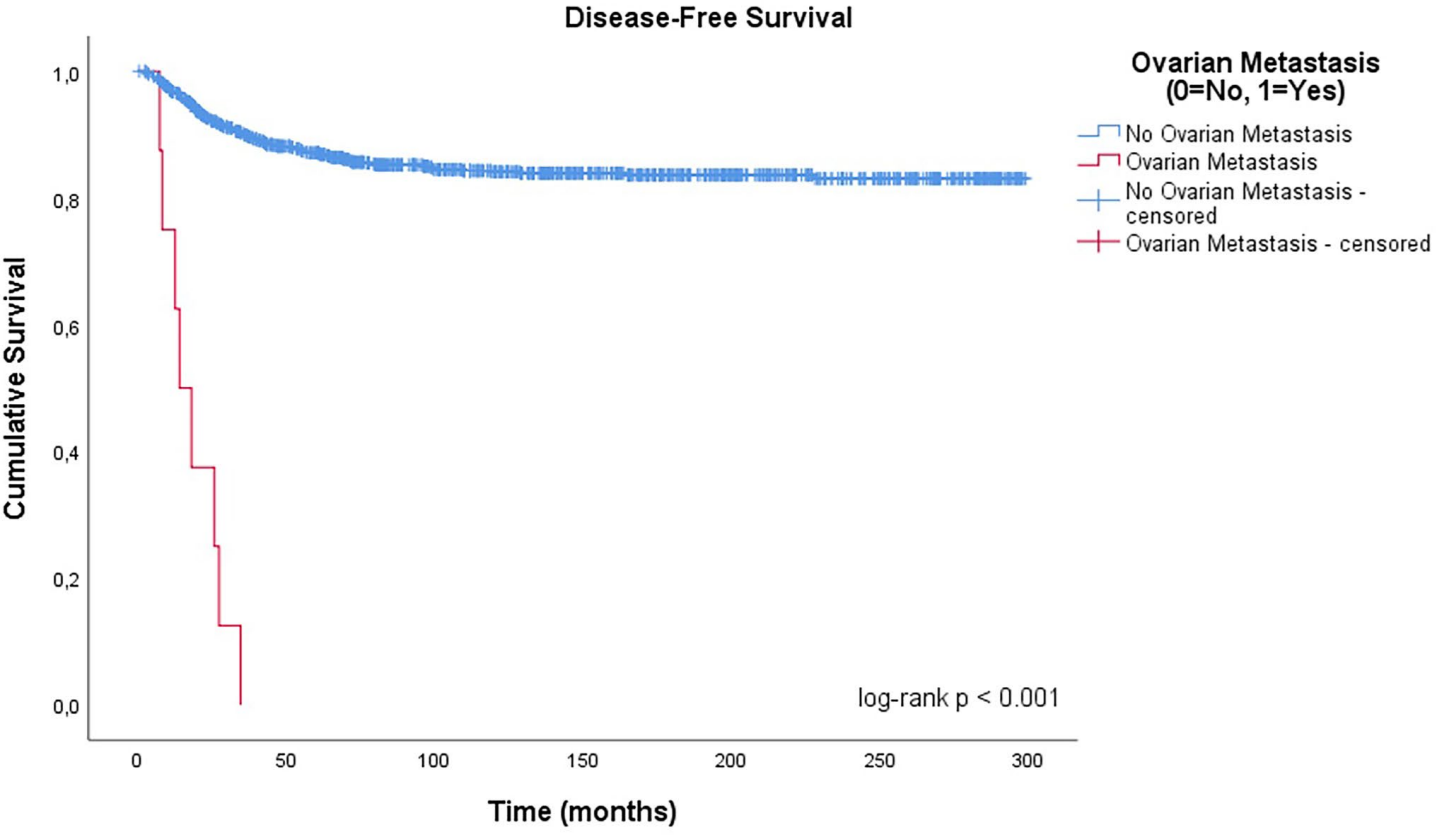
Kaplan–Meier curve for disease-free survival stratified by ovarian metastasis status. Survival differed significantly between groups (log-rank *p* < 0.001)

All 8 patients with ovarian metastases experienced recurrence, whereas 137 of 975 patients (14.1%) in the non-metastatic group had disease recurrence. DFS was significantly reduced in patients with ovarian metastasis (100% vs. 14.1% recurrence rate; log-rank *p* < 0.001; Fig. 3).

## Discussion

### Principal findings

In this large population-based cohort of 983 surgically treated cervical cancers with TNM stages pT1a1–pT2b, we identified a very low overall incidence of ovarian metastasis (0.8%). Only adenocarcinoma histology remained a statistically significant predictor (OR ≈ 10), whereas age, lymphovascular invasion, lymph node status, and pT stage were not associated with ovarian metastasis in this data set. Patients with ovarian metastasis experienced markedly inferior DFS and OS.

### Comparison with the literature

Our incidence is almost identical to the 0.9% reported by Kasamatsu et al. in 1 695 early-stage cases [11] and lies at the lower end of the 1.5–3.6% range summarized in a recent review of > 18 000 patients [12]. Consistent with prior studies, adenocarcinoma conveyed the greatest risk: meta-analyses place the incidence between 2 and 12% depending on stage and series [5, 7], closely matching our 2.6% (6/232).

Several multicentre studies and meta-analyses found pelvic lymph node involvement (PLNI) and lymphovascular space invasion (LVSI) to increase the odds of ovarian metastasis fivefold to tenfold [13–15]. In contrast, neither L status nor N status reached significance in our registry. The most plausible explanation is low event number (*n* = 8), which limits statistical power; two of the eight metastatic cases were LVSI-positive (25%), mirroring published proportions, but confidence intervals remain wide.

Notably, survival outcomes were poor in our metastasis group despite the administration of curative adjuvant therapy in most cases. This aligns with prior studies suggesting that ovarian involvement reflects aggressive tumor biology and is associated with significantly reduced survival, even after multimodal treatment [5, 13, 15]. Previous studies consistently report that patients with ovarian metastases commonly receive adjuvant radiotherapy or chemoradiation; however, most do not provide precise treatment rates. In contrast, our population-based analysis quantified therapy use across both metastatic and non-metastatic groups, offering novel insight into real-world treatment patterns. Few population-based analyses to date have included detailed treatment data, making our findings particularly robust in confirming metastasis as an independent marker of adverse prognosis [10]. The poor prognosis despite adjuvant therapy may reflect aggressive tumor biology or intrinsic resistance of ovarian metastases to conventional therapy.

### Clinical implications

Our findings are consistent with current recommendations that often favor ovarian preservation in squamous cell carcinoma when adjuvant radiotherapy is not planned, although confirmation in larger datasets would be valuable. In adenocarcinoma, the absolute risk was < 3% in our cohort—lower than in earlier series—yet the small event count precludes firm conclusions. Because metastasis was associated with worse survival in our study, careful intra-operative assessment—including, where appropriate, frozen-section analysis—may warrant consideration, but this suggestion should be validated prospectively. Although absolute risks were very low (0.15% for squamous carcinoma, 2.6% for adenocarcinoma), this information is clinically important for counseling but should not be overinterpreted given the small number of events.

For fertility- or menopause-sparing surgery, individualized counseling appears warranted; women with adenocarcinoma could be informed that the relative risk was ~ tenfold higher in our dataset, while the observed risk in squamous histology was very low. Larger studies are needed to refine these estimates.

Most patients with ovarian metastasis received protocol-based adjuvant therapy with curative intent, including combined chemoradiation in the majority. Despite this, survival outcomes remained markedly inferior, suggesting that the presence of ovarian metastasis itself drives prognosis more than treatment variation. Conversely, over half of the non-metastatic cohort received no adjuvant therapy, supporting the oncologic safety of ovarian preservation in low-risk patients. These findings reinforce the importance of individualized decision-making and risk stratification when planning fertility- or menopause-sparing surgery.

The 42% adjuvant therapy rate observed in non-metastatic patients aligns with prior literature, which reports rates between 30 and 50% for surgically treated FIGO stage IA1–IIB cervical cancer depending on pathologic risk factors [4, 5, 8]. This confirms that our cohort reflects standard treatment patterns and highlights that ovarian preservation in early-stage cervical cancer is often considered when adjuvant therapy is unlikely to be required.

### Strengths and limitations

Strengths include a state-wide, population-based registry, uniform bilateral oophorectomy allowing histological confirmation, and long median follow-up of 105 months. Limitations are the retrospective design and the very low number of metastatic events, which precluded a fully adjusted multivariable model and widened confidence intervals. The low number of events restricted formal multivariable survival modeling incorporating treatment variables. Given the rarity of ovarian metastasis in our cohort, we also acknowledge the potential limitations of standard logistic regression under rare event conditions. However, model convergence and confidence intervals were acceptable, and more specialized methods such as exact logistic regression or penalized likelihood estimation may be considered in future studies to validate these findings. The extremely low number of events (*n* = 8) severely limited statistical power, resulting in wide confidence intervals and instability of regression estimates. Multivariable survival analysis incorporating treatment parameters could not be performed. Prognostic conclusions, therefore, remain tentative, and the study should be interpreted as descriptive.

### Conclusion and future directions

Ovarian metastasis from early-stage cervical cancer is rare (0.8%), largely confined to adenocarcinoma, and associated with poor survival despite adjuvant therapy. While adenocarcinoma histology appears to confer elevated risk, the small number of events and exclusion of patients with ovarian preservation limit generalizability. These findings should be interpreted as descriptive and hypothesis-generating, supporting individualized surgical decision-making and informing future larger pooled analyses.

## Data Availability

No datasets were generated or analyzed during the current study.
